# Necrotizing Fasciitis of the Upper Limb: Optimizing Management to Reduce Complications

**DOI:** 10.3390/jcm11082182

**Published:** 2022-04-13

**Authors:** Simone La Padula, Rosita Pensato, Antonio Zaffiro, Oana Hermeziu, Francesco D’Andrea, Chiara Pizza, Jean Paul Meningaud, Barbara Hersant

**Affiliations:** 1 Department of Plastic and Reconstructive Surgery, Università Degli Studi di Napoli Federico II, Via Pansini 5, 80131 Naples, Italy; rositapensato@gmail.com (R.P.); antoniozaffiro14@gmail.com (A.Z.); profdandrea@gmail.com (F.D.); 2 Department of Plastic, Reconstructive and Maxillo Facial Surgery, Henri Mondor Hospital, University Paris XII, 51 Avenue du Maréchal de Lattre de Tassigny, 94000 Creteil, France; oanaher@gmail.com (O.H.); chiapiz@gmail.com (C.P.); meningaud@me.com (J.P.M.); barbara.hersant@gmail.com (B.H.)

**Keywords:** necrotizing fasciitis, hand infection, soft tissue infection, hand reconstruction, upper limb infection, upper limb necrotizing fasciitis

## Abstract

Background: Necrotizing fasciitis (NF) is a severe, potentially life-threatening condition. The aim of this study is to identify strategies aimed at reducing complications in patients with NF of the upper limb. Methods: We conducted a retrospective study on patients admitted to our Unit for suspected NF of the upper limb. The analyzed data included patient characteristics, delay before primary care, clinical and biological signs upon arrival, pathogens involved, and the rate of amputations and mortality. Results: A total of 21 patients presented with confirmed necrotizing bacterial dermohypodermitis-NBDH with NF (NBDH-NF) affecting the upper limb. The mean delay between the onset of symptoms and the clinical examination in the Emergency Dermatology Unit was 48 h (range: 6 to 72 h). The mean delay between admission and primary surgery was 150 min (range: 60 min to 280 min). No amputations were performed. All patients were alive one year after the first surgical procedure. Conclusions: Our study demonstrated that it is possible to reduce mortality and morbidity rates in NF of the upper limb. Timely diagnosis and early treatment and a multidisciplinary medico-surgical dedicated team providing care can significantly modify the outcomes. Early surgical debridement is the most important factor affecting the prognosis of these infections.

## 1. Introduction

Necrotizing bacterial dermohypodermitis and necrotizing fasciitis (NBDH-NF) are severe infections caused by necrosis of the dermis and hypodermis (necrotizing bacterial dermohypodermitis-NBDH), that may extend to superficial aponeurosis (necrotizing fasciitis-NF) and to the muscle (myositis). The clinical spectrum of NBDH-NF varies in severity, ranging from cutaneous or isolated subcutaneous necrosis to signs of septic shock.

The broad diversity of clinical presentation and severity is likely to contribute to a detrimental delay in its diagnosis and treatment.

NBDH-NF is associated with high morbidity. While the mortality risk is significant, it varies depending on the study [1,2,3,4,5,6,7,8,9,10,11,12,13,14,15,16]. NBDH-NF requires multidisciplinary management (emergency medicine, intensive care medicine, anesthesiology, plastic surgery, general surgery, dermatology, radiology, and microbiology).

However, NF is rare, and its diagnosis is challenging, with a 50% rate of misdiagnosis upon admission of patients [1]. The difficulty in coordinating the work of different healthcare professionals may cause delays in care. The timing of the primary debridement is crucial. Timely diagnosis and treatment performed by a specialized team can significantly reduce the morbidity and mortality associated with NBDH-NF [14,15,16]. NBDH-NF affects the lower limbs [16] more commonly than the upper extremities [17]. Most studies on NBDH-NF do not distinguish between lower and upper limbs. There are no data in the current literature on delay in the primary surgery on the NBDH of the upper limb [17,18,19,20,21]. The sequelae of NBDH of the upper limb are major disability and functional impairment, with an average amputation rate of up to 25% [22]. The authors conducted a retrospective study on patients diagnosed with NBDH-NF of the upper limb who received care in the Henri Mondor reference center.

## 2. Materials and Methods

The authors conducted a retrospective study on the management of patients treated in the Reconstructive Surgery Department of the Henri Mondor Hospital from May 2014 to May 2016 for suspected upper limb NBDH-NF. Only patients who were diagnosed with NBDH-NF based on histopathology were included in the study. The primary data studied were the patients’ general characteristics, the delay between the onset of clinical signs and the time of attendance at the Emergency Dermatology Unit, the delay before the primary surgery (delay between dermatology referral and surgical debridement), the clinical and biological signs upon arrival, the microbiology, the amputation, and the mortality rate. We also looked into the reconstruction techniques used to resurface the upper limb when the infection had cleared. The study was approved by our institutional review board (IRB). All subjects were informed about the study’s purpose and gave their consent for data analysis and publication.

### 2.1. Standardized Management of Care of the NF Group of the Henri Mondor Institution

The NF Group consists of a multidisciplinary team including dermatologists, critical care physicians and plastic surgeons specializing in the management of necrotizing soft tissue infections. An innovative feature of this setting is that patients presenting with signs and symptoms of NBDH-NF are directly referred to the Emergency Dermatology Unit. Immediately after the diagnosis, the plastic surgery team (on call 24/7) carries out the primary surgical debridement. In selected cases, an emergency Magnetic Resonance Imaging (MRI) scan can be performed to confirm a clinical diagnosis. This emergency setting minimizes any potential delay between the diagnosis and the primary surgery.

All the cases included in our study were started upon diagnosis and prior to any surgical procedure on broad-spectrum parenteral antibiotic therapy with 4 g of piperacillin–tazobactam 4 times daily and 600 mg of clindamycin 4 times daily. An immediate IV dose was administered. Based on their hemodynamic status, stable patients were admitted to the plastic surgery ward following primary surgery; unstable patients were sent to the intensive care unit.

### 2.2. Surgical Protocol

Surgery was always performed under general anesthesia. All the patients were stabilized prior to surgery, to ensure optimal hemodynamic during the procedure. Tourniquet was not applied during surgery in order to better assess tissue viability and identify devascularized tissue requiring excision. In all cases, the skin, subcutaneous tissue and fascia were removed “en bloc”. The primary surgical debridement routinely started at the entry point, followed by the exploration of any other affected area. Any area of superficial aponeurotic tissue detaching spontaneously, or lacking tonicity, was considered necrotic and was systematically excised. The primary muscle belly was also checked: if not viable, it was excised. The wound was generously irrigated with saline solution. In some cases, a combination of saline solution and diluted antiseptic (povidone-iodine, Betadine^®^ Mylan, Mérignac, France) was used. A final wash-out with hydrogen peroxide was then performed.

If the hand or fingers were affected, surgeons wore binocular magnifying loupes during necrotic tissue excision in order to avoid iatrogenic injuries to nerves and blood vessels. Multiple microbiology and histopathology samples were harvested from the affected areas and analyzed to identify the specific pathogens and to confirm the diagnosis.

When healthy and viable tissue was obtained after debridement, Algosteril (containing wound-healing stimulating substances) dressings were applied to the wound. Dressings were changed daily.

## 3. Results

Between May 2014 and May 2016, 193 patients attended our Institution for suspected NBDH-NF. A total of 21 patients (17 men and 4 women) were diagnosed with confirmed NBDH-NF of the upper limb and showed signs of necrosis of the dermis, hypodermis (necrotizing bacterial dermohypodermitis-NBDH), and superficial aponeurosis (necrotizing fasciitis—NF). The patients’ mean age was 51 ± 9.3 years. Upon hospitalization, 9 out of 21 (42%) presented with co-morbidities. The number of co-morbidities was, on average, 2 per patient (range: 0 to 4). The most common co-morbidities included obesity (7/21) and diabetes (6/21). Thirteen patients were smokers (61.9%). The mean delay between the onset of signs and symptoms and the Emergency Room dermatology examination was 48 h (range: 6 to 72 h). The mean delay between admission and the primary surgery was 150 min (range: 60 to 280 min). Seventeen patients reported a primary superficial cutaneous lesion or other cutaneous lesions of the hand (Table 1).

Locoregional signs included swelling/increase in the size of the limb, extreme redness and warmth, and pain out of proportion compared to the clinical presentation (15/21); in selected cases, signs such as an obvious cutaneous necrosis (4/21) and sub-cutaneous emphysema (2/21) were also present. A total of 13 patients were feverish upon admission; 3 patients showed signs of severe sepsis upon admission, including hypotension and tachycardia; the remaining patients were stable.

In 18 cases, clinical signs were sufficient to diagnose NBDH; in 3 cases, an emergency MRI scan was necessary to confirm diagnosis. In nearly all the cases the following findings were observed: an increase in white blood cells (average 15.8 × 10^9^/L ranging from 10.8 to 29.2), an increase in CRP (average 197 mg/L ranging from 39 to 430), and an increase in lactate levels (average 3.2 mmol/L ranging from 2.9 to 14.3). At the time of the primary surgery, the affected areas were: hand and wrist for 3 patients; hand, wrist and forearm for 10 patients; hand, wrist and forearm extending to the arm for 8 patients.

The patients had suffered trauma or injury to a finger (entry point) hours or a day before the onset of symptoms and signs of infection.

**Figure 1:**
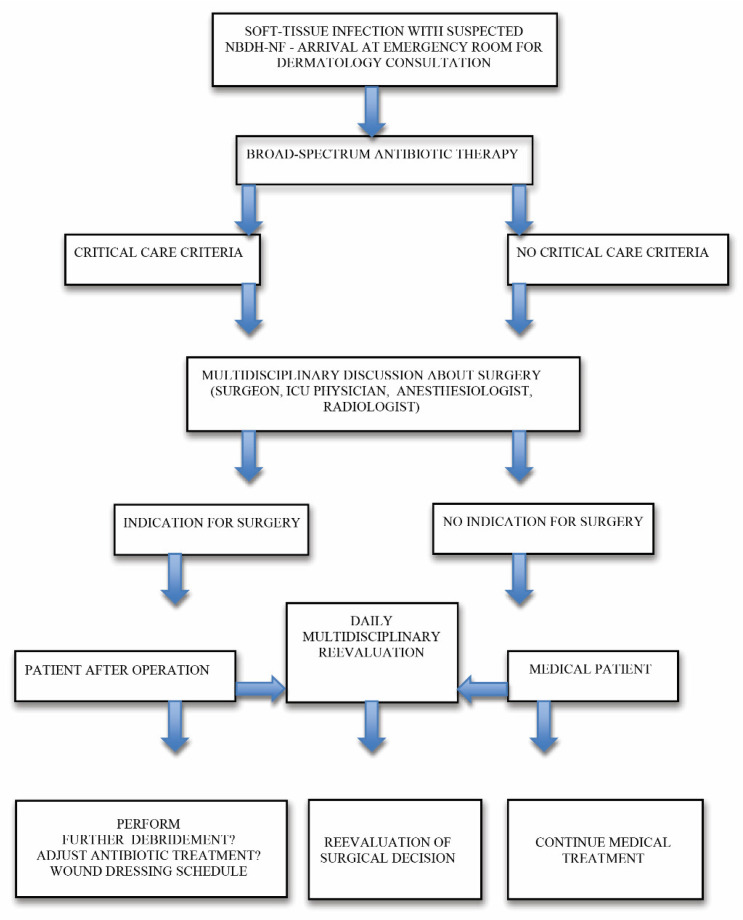
Authors’ algorithm for upper limb NF management.

In 20 cases (95%), primary surgery was beneficial and promoted a significant improvement in the local and general condition. Between 2 and 4 days after the primary surgery, 12 patients required secondary debridement in the operating room, and 9 patients had revision surgery one week after the first procedure (Table 1). The authors have developed an algorithm for upper limb NBDH-NF management (Figure 1), based on their clinical experience and findings.

### 3.1. Microbiology

Four microbiology samples were harvested intraoperatively from each patient on each affected area, in accordance with our Institution’s protocol for bacteriological, parasitological, fungal and histopathological analyses. Five cases of Type I NF were identified based on the Giuliano classification (polymicrobial infection). The following bacterial spectrum was isolated: Methicillin-resistant Staphylococcus aureus (MRSA) and *P. Aeruginosa* in one case; coagulase-negative methicillin-sensitive *Staphylococcus* and Enterococci in another case; *Staphylococcus aureus* and *Pseudomonas aeruginosa* in two cases; and *M. morganii* and *E. faecalis* in another case. Sixteen cases of Type II NF were identified: a monomicrobial infection sustained by group A beta-hemolytic streptococci (GABHA) was detected (Table 2).

The clinical condition of these 16 patients changed very rapidly (within 24 h of the first appearance of symptoms), with distant progression of the erythema and spreading of other local signs distally and proximally to the affected area; only one patient developed septic shock, one hour after admission. For each patient, antibiotic treatment was adjusted based on the results of antibiotic susceptibility tests.

**Table 2:** Pathogenic agents.

NF Type I (Polymicrobial) *n* = 5	One Case: MRSA and *P. Aeruginosa*
	One case: methicillin-sensitive *Staphylococcus* and Enterococci
	Two cases: *Staphylococcus aureus* and *Pseudomonas aeruginosa*
	One case: *M. morganii* and *E. faecalis*
NF Type II, monomicrobial *n* = 16	16 cases: GABHA
Muscle tissue affected (myositis)	2 cases: GABHA NBDH-NF (2/16)

MRSA: methicillin-resistant staphylococcus aureus. GABHA: group A beta-hemolytic streptococci. NBDH-NF: Necrotizing bacterial dermohypodermitis-necrotizing fasciitis.

### 3.2. Morbidity, Amputations and Mortality

No amputations were performed. In two patients, muscular tissue was affected and multiple segments of the brachioradialis and flexor carpi radialis muscle were excised. Nine patients required physical therapy sessions to promote functional recovery (an average of 60 sessions, ranging from 20 to 90). All patients were alive one year after the first surgical procedure (Table 3).

### 3.3. Reconstructive Surgery

**Figure 2:**
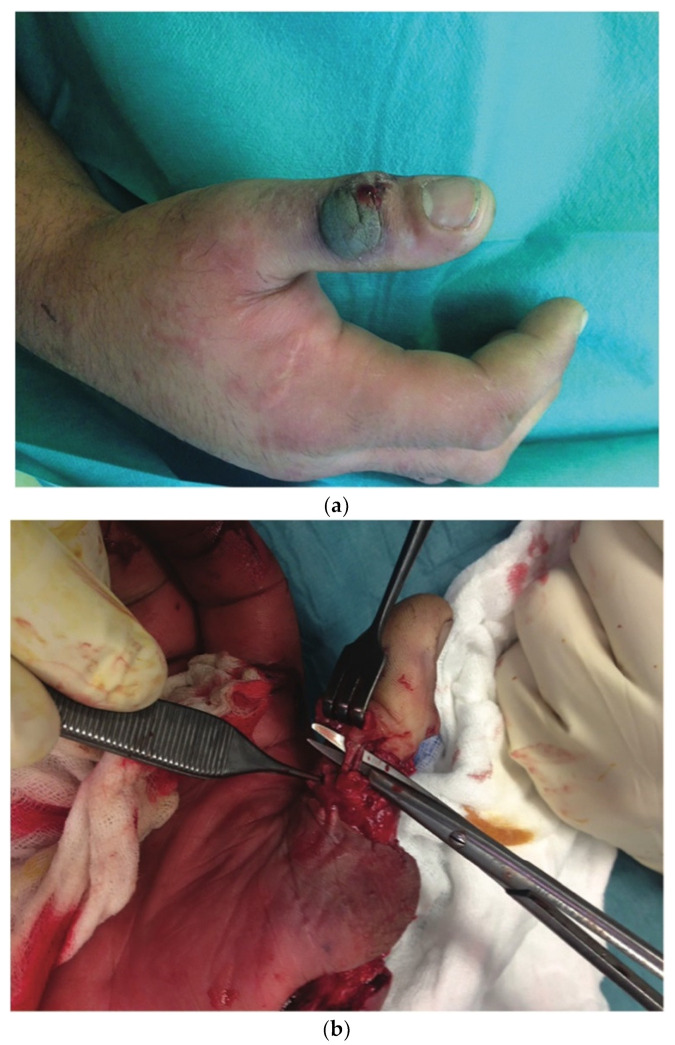

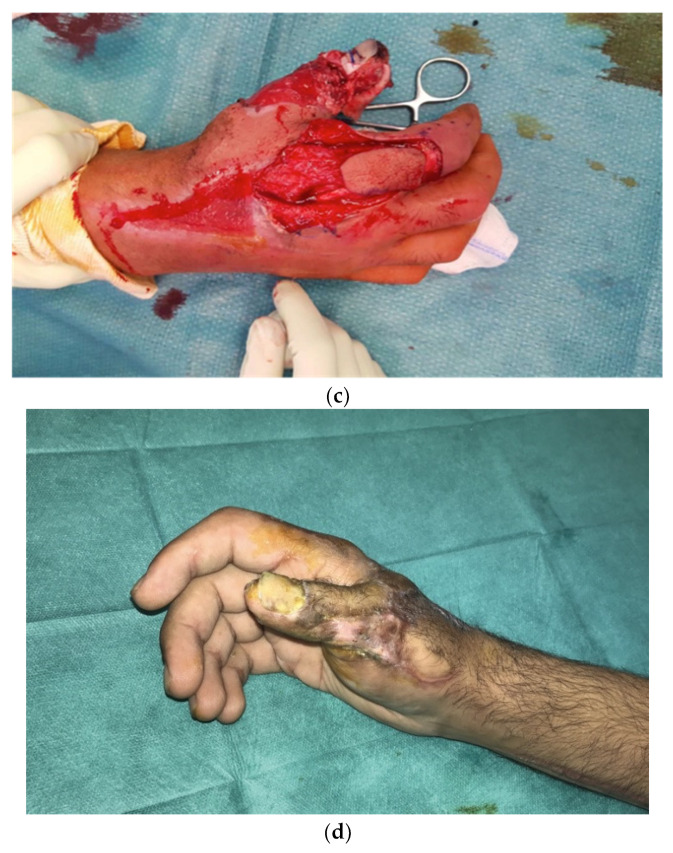
A 27-year-old patient presented with the clinical signs of necrotizing fasciitis of the right hand following an injury involving a wrought-iron handle 48 h earlier (**a**). Exploration of the vascular pedicles of the right thumb had revealed contracted vessels surrounded by infected tissue; a precautionary debridement was performed to preserve the vascular pedicles (**b**). The necrotic areas and all the non-viable tissue of the right thumb were debrided, with excision of the long extensor tendon of the thumb. The IP joint was opened, revealing the presence of pus; the cartilage was then resected until healthy, vascularized bone was reached. Following liberal irrigation of the articulation and tissues using Betadine^®^, we ensured arthrodesis of the thumb IP joint with two pins using X-ray guidance. To cover the loss of soft tissue on the dorsal side of the thumb, leaving the IP joint exposed, it was decided to perform a kite-shaped dorsal metacarpal artery flap. The flap was drawn on the dorsal side of the first phalange of the second finger, going from the extension fold of the PIP, up to the metacarpophalangeal joint and laterally to the mediolateral line (**c**). The flap donor site and the flap pedicle were covered by a full-thickness skin graft removed from the inner surface of the right arm. Six months later, the result was satisfactory, with good functional recovery and articular mobility (**d**).

The mean delay for grafting and wound resurfacing was 6 weeks (42 ± 7 days). In 15 cases, a split-thickness skin graft taken from the thigh was used to resurface the arm and forearm: 2 of these 15 patients had a full- thickness skin graft harvested from the medial aspect of the arm and transferred to the dorsal aspect of the hand and fingers. Only one patient required resurfacing of the dorsal aspect of the interphalangeal joint of the thumb. In this case, a secondary procedure based on a kite-shaped dorsal metacarpal artery flap was performed (Figure 2). For the cases (*n* = 3) that had only required limited superficial debridement (up to 4 cm^2^ in our study), healing by secondary intention was promoted and centripetal epidermalization took place (Table 4).

**Table 4:** Reconstructive surgery.

Patients Receiving Thin Skin Grafts to Cover Loss of Soft Tissue	*n* = 15 (Covering Soft Tissue of the Forearm and Arm)
**Patients receiving a full thickness skin graft** **to cover loss of soft tissue**	*n* = 2 (2 patients from among the previous 15 cases), a full thickness skin graft was used to cover the back of the hand and the dorsal side of the thumb and second finger respectively.
**Flaps**	*n* = 1 kite-shaped flap to cover loss of soft tissue of the dorsal side of thumb
**Healing by secondary intention**	*n* = 3 mean delay for healing 98 days

## 4. Discussion

Necrotizing fasciitis (NF) is a severe and potentially life-threatening condition. In most cases, the diagnosis is based on clinical signs, but sometimes, the differential diagnosis with other conditions may not be easy, especially with Pyoderma gangrenosum. However, the latter is ulcerative skin necrosis, and an experienced team can recognize the clinical differences between these lesions. If doubts remain and the patient shows no signs of septic shock, imaging (MRI and/or CT scan) and laboratory criteria can be helpful. Fascial enhancement, fascial edema and fascial gas are signs of NF detected at the CT. However, the diagnostic accuracy of magnetic resonance imaging (MRI) can recognize subtle signs of NF, potentially allowing for earlier diagnosis. The Laboratory Risk Indicator for Necrotizing Fasciitis (LRINEC) score is a diagnostic clinical decision-making instrument validated for differentiating NSTI from other soft tissue infections [17]. LRINEC utilizes six laboratory serum parameters, including white blood cell (WBC) count, hemoglobin, sodium, glucose, creatinine, and C-reactive protein. A score of 6 (the traditional threshold for diagnosis of NSTI) indicates a “moderate” risk of NSTI (50–75% probability), whereas a score of 8 indicates a “high” risk (greater than 75% probability).

If the patient is in shock upon arrival, a decision must be made immediately, since there is no time for imaging. In this case, a small incision can be made to check the underlying tissues.

If the subfascial plane is easily detached with a finger, it is almost certainly necrotizing fasciitis, and the patient is immediately transported to the operating room.

To date, very few studies have assessed the management of patients suffering from NBDH-NF of the upper limb and conducted the analysis of specific data [18,19,20,21,22,23,24,25]. Significant morbidity and mortality were described, with an amputation rate of up to 25% and a mortality rate of up to 35.7% [19,20,21,23,25]. Although the upper limb is less frequently affected, pathological scarring sequelae and functional impairment must be taken into account [22]. The challenge is to ensure timely recognition and management of this condition. Imaging tests may facilitate diagnosis, but they should never delay surgery.

A recent investigation illustrated the variability in the management of NF. Delays in the primary surgery are common. Usually, such delays occur due to late diagnosis, or late formal surgical indication. Delays may also be due to the absence of specialized care facilities [26]. All these factors contribute to reported delays in the primary surgery. These data suggest that creating a dedicated care unit specializing in the management of NFs could significantly reduce mortality and morbidity rates.

Our results (0% amputation and mortality rates) may be explained by multiple factors: timely diagnosis and management carried out by a dedicated NBDH-NF team; priority access to the operating room when surgery is indicated.

Reconstructive surgery resurfaces tissue defects and reduces the amputation rates thanks to salvage flaps that protect Noble tissues exposed after primary surgery.

Strict post-operative monitoring and physical therapy are required to improve and accelerate the functional recovery of the affected areas.

However, even in very severe cases with massive loss of functional structures, the extremity and its function can be restored by microsurgical and reconstructive procedures such as flap transfer, autologous nerve transplantation/neurotization and functional muscle transfer techniques, either as a combined or a staged procedure.

The authors have developed an algorithm for upper limb NBDH-NF management (Figure 2), based on their clinical experience and findings.

The authors recommend not to amputate in primary surgery. This strategy is aimed at reducing the morbidity rates (amputation, loss of hand function) associated with upper extremity NBDH-NF. If a joint is exposed after primary debridement, a salvage flap can often obviate the need for amputation.

The use of surgical binocular magnifying loupes is recommended during the excision of infected and necrotic tissue to avoid iatrogenic injuries to nerves and blood vessels.

If the tissue surrounding a digital vascular pedicle is affected, it may provoke thrombosis or constrictions. The use of vasodilators and techniques to promote hand rewarming can often improve vasoconstriction and avoid primary amputation [27,28,29].

Tissue viability should always be checked; in case of doubt, further surgical explorations under general anesthesia are recommended to ascertain the viability of the tissues, to check that the infection has not progressed and to perform additional excisions if necessary.

In all cases, patients should be monitored daily, and additional debridement performed if needed.

Mortality and morbidity rates from NBDH of the upper limb may be reduced through timely and prompt diagnosis and treatment carried out by a dedicated multidisciplinary medical–surgical team. Initial severe sepsis and underlying co-morbidities may worsen prognosis. Early surgical debridement crucially affects the prognosis of these infections.

## Figures and Tables

**Table 1:** Patient data.

Patients *n* = 21	17 Men–4 Women
**Affected areas**	Hand and wrist *n* = 3 Hand, wrist and forearm *n* = 10 Hand, forearm and arm *n* = 8
**Point of entry**	Hand (*n* = 17) Wrist (*n* = 2) Unidentified (*n* = 2)
**Mean age**	51 ± 9.3
**Co-morbidities**	
- Obesity - Diabetes - Smoking	*n* = 7 (33%) *n* = 6 (28.5%) *n* = 13 (61%)
**Mean delay between the appearance of symptoms and hospitalization (hours)**	48 ± 5.6 (6–72)
**Mean delay between hospitalization and first operation (minutes)**	150 ± 23 (100–290)
**Patients requiring only one revision surgery**	12
**Patients requiring two revision surgeries**	9

**Table 3:** Main series of NBDH-NF of the upper limb.

Series	Cases (Number of Patients)	Amputation Rate %	Mortality Rate %
Schecter W. 1982 [20]	33	6	9
Bleton R. 1991 [21]	12	0	16.5
Gonzalez MH. 1996 [23]	12	25	0
Cheng NC. 2008 [19]	14	0	35.7
Hankins C 2008 [24]	31	3.2	0
Henri Mondor NF Group	21	0	0
